# Differences in Center for Epidemiologic Studies Depression Scale, Generalized Anxiety Disorder-7 and Kessler Screening Scale for Psychological Distress Scores between Smartphone Version versus Paper Version Administration: Evidence of Equivalence

**DOI:** 10.3390/ijerph20064773

**Published:** 2023-03-08

**Authors:** Kazuki Hirao, Hyono Takahashi, Natsuki Kuroda, Hiroyuki Uchida, Kenji Tsuchiya, Senichiro Kikuchi

**Affiliations:** 1 Graduate School of Health Sciences, Gunma University, Maebashi 371-8514, Japan; 2 Department of Occupational Therapy, Faculty of Medicine, Gunma University, Maebashi 371-8514, Japan; 3 Department of Rehabilitation, Kurashiki Heisei Hospital, Kurashiki 710-0826, Japan; 4 Department of Rehabilitation, Faculty of Health Sciences, Nagano University of Health and Medicine, Nagano 381-2227, Japan

**Keywords:** patient-reported outcomes, electronic, depression, anxiety, smartphone

## Abstract

The use of electronic patient-reported outcomes has increased recently, and smartphones offer distinct advantages over other devices. However, previous systematic reviews have not investigated the reliability of the Center for Epidemiologic Studies Depression Scale (CES-D), Generalized Anxiety Disorder-7 (GAD-7), and Kessler Screening Scale for Psychological Distress (K6) when used with smartphones, and this has not been fully explored. This study aimed to evaluate the equivalence of the paper and smartphone versions of the CES-D, GAD-7, and K6, which were compared following a randomized crossover design method in 100 adults in Gunma, Japan. Participants responded to the paper and smartphone versions at 1-week intervals. The equivalence of paper and smartphone versions was evaluated using the intraclass correlation coefficient (ICC_agreement_). The mean participant age was 19.86 years (SD = 1.08, 23% male). The ICC_agreements_ for the paper and smartphone versions of the CES-D, GAD-7, and K6 were 0.76 (95% confidence interval [CI] 0.66–0.83), 0.68 (95% CI 0.59–0.77), and 0.83 (95% CI 0.75–0.88), respectively. Thus, the CES-D and K6 scales are appropriate for use in a smartphone version, which could be applied to clinical and research settings in which the paper or smartphone versions could be used as needed.

## 1. Introduction

The use of patient-reported outcomes (PROs) is neccessary because of several advantages [1,2,3]. Previous studies have shown that the use of PROs to systematically monitor patient symptoms improves patient–physician communication, symptom oversight, and gaps in patient health, quality of life, and clinician perception of symptoms [1,2,3]. PROs are also widely used in the mental health field, and mental health clinicians suggest that the use of PROs in patient consultations can help in making treatment decisions and severity assessment [4,5]. Depressive symptoms, anxiety, and psychological distress are particularly common in the field of mental health, and it has been indicated that these symptoms may coexist and affect each other [6,7,8,9,10,11,12]. As a result, it is crucial to thoroughly evaluate utilizing PRO not just one symptom but also depressed symptoms, anxiety symptoms, and psychological distress. Currently, many PROs exist to measure depressive and anxiety symptoms and psychological distress. For example, the Center for Epidemiologic Studies Depression Scale (CES-D) [13,14], Generalized Anxiety Disorder-7 (GAD-7) [15,16], and Kessler Screening Scale for Psychological Distress (K6) [17,18] are widely used PROs to measure depressive and anxiety symptoms. The CES-D is a 20-item PRO developed to assess depressive symptoms in both clinical and nonclinical settings [13,14]. The GAD-7 is a 7-item PRO used in screening for generalized anxiety disorder and other anxiety disorders, such as panic disorder, social anxiety disorder, and post-traumatic stress disorder [15,16]. K6 is a 6-item PRO developed to measure psychological distress [17,18]. These PROs can be answered in a short time and are easy to grade [10,14,16,17,19,20,21]. In addition, they were translated in many languages, and their psychometric properties, including reliability and validity, have been reported [10,13,14,15,16,17,18,22,23,24,25,26,27,28,29]. The CES-D, GAD-7, and K6 have been translated into Japanese, and their reliability and validity have been examined in several studies [10,13,17,26,29,30]. Given these advantages, CES-D, GAD-7, and K6 are widely used in both clinical and epidemiological studies and diagnostic screening in the local general population [19,20,31,32]. Importantly, these PROs are also inevitably used as electronic patient-reported outcomes (ePROs) as increasingly more studies in the mental health field use the Internet [33,34,35,36,37,38].

Compared with paper-based PROs, ePROs minimize errors in score calculation and data entry and missing data, facilitating reliable analysis and reporting of PRO data [39,40,41,42]. Previous studies have also suggested that patients prefer ePROs to paper-based PROs, and by using ePROs, patients may disclose more sensitive information than paper-based PROs [42,43,44,45,46,47,48]. As a result, making the CES-D, GAD-7, and K6 available as ePROs, which have reliability and validity and are employed in many countries, will not only make these instruments easier to use for participants, researchers, and healthcare professionals but may also decrease administrative burden and avoid missing data. Smartphones are playing an increasingly important role in capitalizing on these potential benefits of ePRO use in clinical and research settings.

Currently, many devices are being utilized for ePROs [42]. Among them, smartphones offer distinct advantages over other devices for the use of ePROs. Smartphone users are increasing worldwide, and most people carry their smartphones with them at all times [39,49]. In addition, more people are using smartphones than personal computers (PCs) to access the Internet [49]. Therefore, smartphones will enable PROs in a more real-time manner than PCs or tablets. Moreover, several studies point to the value of employing smartphones as ePRO devices [39,50,51]. However, to the best of our knowledge, no study has confirmed the equivalence of the electronic and paper versions of the K6 and GAD-7. However, several previous studies have confirmed the equivalence of the electronic and paper versions of the CES-D [52,53]. Contrarily, a previous systematic review did not verify the equivalence between the smartphone version of the CES-D and the paper version of the CES-D [52,53]. As a result, it is unlikely that the reliability of the CES-D, GAD-7, and K6 when applied to smartphones has been sufficiently researched. [38,46,54]. The International Society for Pharmacoeconomics and Outcomes Research (ISPOR) guidelines suggest that differences in how ePRO and original PRO questions are presented may adversely affect the reliability and validity [42]. In addition, and of particular importance, the reliability and validity of ePROs may be affected by the type of device used, e.g., PCs or tablets [42,46]. Because of these issues related to the transition from the original PRO to ePRO, the ISPOR guidelines need cognitive debriefing and usability testing to be conducted for minor changes (i.e., from circling the answer to touching the answer on the screen, etc.) when changing from PRO to ePRO. Moderate changes (i.e., need to scroll the screen, change font size, etc.) show the need to perform reliability measures (e.g., intraclass correlation coefficient), while large changes (e.g., concerning response choices or item wording) demonstrate the need for full psychometric testing [42]. Therefore, the ePRO and the original PRO should be compared on a device-by-device basis, and whether they are equivalent, if not superior, must be verified. Therefore, this study aimed to examine the measurement equivalence of the paper and smartphone versions of CES-D, GAD-7, and K6 based on ISPOR guidelines.

## 2. Materials and Methods

### 2.1. Study Design

**Figure 1:**
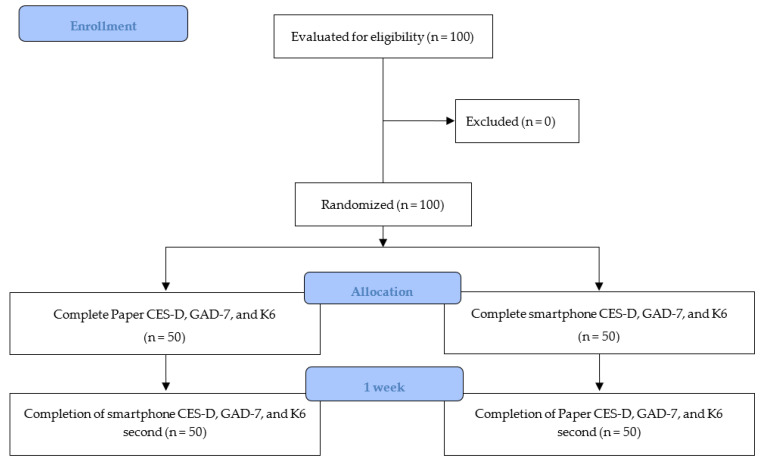
Flowchart of trial.

This study was conducted using a randomized crossover design to assess the format equivalence of the paper and smartphone versions of the CES-D, GAD-7, and K6. Figure 1 depicts the process of the randomized crossover design used in this investigation. The study was conducted in accordance with ISPOR guidelines [42] and was approved by the Ethical Review Board for Medical Research Involving Human Subjects of Gunma University (Approval no. HS2022-109). Written informed consent was obtained from each participant before study participation.

### 2.2. Participants and Procedure

The study participants were recruited between October 2022 and December 2022 from Gunma University in Gunma, Japan. The recruitment was made by posting posters at Gunma University. Study participation was also encouraged via e-mail and social networking services. Individuals aged ≥18 years who were native Japanese speakers and had a smartphone were considered eligible for this study. Participants who met the eligibility criteria were asked to complete the CES-D, GAD-7, and K6 scales (paper and smartphone versions) after answering demographic information (age and sex) and lifestyle characteristics (i.e., drinking, exercise, and smoking habits). The order in which the PROs were filled out (paper version first or smartphone version first) was randomly determined. To reduce potential recall and carryover effects, the interval between the completion of the two questionnaires was 1 week.

### 2.3. Randomization

Participants were randomly assigned in a 1:1 ratio to complete either the paper version first or the smartphone version first before answering the questionnaire (CES-D, GAD-7, and K6). The randomization list was generated by a permuted block method (block size 4) using a computer (Microsoft Excel) by a third party unrelated to the study. The randomization list was sent to the Central Registry Center at Kurashiki Heisei Hospital in Okayama Prefecture, Japan, for random assignment.

### 2.4. Sample Size

The ISPOR guidelines report that 43 participants with no missing data are needed to declare an ICC of ≥0.7 at 80% power and 95% confidence level if the ICC observed in two measurements is expected to be 0.85, using the approximation used by Walter et al. [42,55]. Conversely, the Consensus-based Standards for the Selection of Health Measurement Instruments initiative suggests that a sample size of ≥100 is necessary to obtain statistical power when evaluating test–retest reliability [56]. Taken together, these findings suggest a target sample size of 100 study participants.

### 2.5. Measures

#### 2.5.1. CES-D

The CES-D is a 20-item self-report questionnaire used to measure depressive symptoms [13,14]. Each item has a 0–3 Likert scale (A = <1 day, B = 1–2 days, C = 3–4 days, and D = 5–7 days) with a total score of 0–60. Higher scores indicate high levels of depressive symptoms. Previous studies have reported the reliability and validity of the CES-D score [10,13,14,28,29,57].

#### 2.5.2. GAD-7

The GAD-7 is a 7-item self-report questionnaire used to measure generalized anxiety disorder, on a 0–3 Likert scale (0 = not at all sure, 1 = several days, 2 = over half the days, and 3 = nearly every day) [15,16]. The total scores range from 0 to 21, with higher scores indicating greater anxiety. Previous studies have reported the reliability and validity of the GAD-7 score [15,16,22,23,24,25].

#### 2.5.3. K6

The K6 is a 6-item self-report questionnaire used to measure psychological distress, using a 0–4 Likert scale (0 = none of the time, 1 = a little of the time, 2 = some of the time, 3 = most of the time, and 4 = all of the time) [17,18]. Total scores range from 0 to 24, with higher scores indicating greater psychological distress. Previous studies have reported the reliability and validity of the K6 score [10,17,18,26,27].

### 2.6. Software

Electronic versions of CES-D, GAD-7, and K6 were provided on participants’ smartphones using Google Forms. The questionnaires were presented in the order CES-D, GAD-7, and K6. The questions, answer choices, and order of questions in the electronic version are the same as those in the paper version of the three scales. Each questionnaire was presented on a separate page; however, all the questions for each questionnaire are displayed on the screen. Scrolling down the screen allows the user to move to the next answer. After answering all the questions in the questionnaire, the next questionnaire can be answered by pressing the “Next” button (specifically, the 20 questions in the CES-D are displayed on a single page, and after answering all of them, the “Next” button is pressed to move to the GAD-7 questionnaire page). Participants can select their answers by tapping the radio buttons on the screen. It is not possible to move to the next page without answering a question item or to select two answers to the same question. However, it is possible to change a previous answer by pressing the “Back” button.

### 2.7. Statistical Analysis

In this study, the switch from the paper version to the smartphone version corresponds to the light to moderate adjustment suggested by the ISPOR guidelines [42]. As a result, to confirm the equivalence of each scale between the paper and smartphone versions, the intraclass correlation coefficient (ICC_agreement_) and its 95% confidence interval were calculated based on the two-way random-effects model, one of the most commonly used statistical measures in equivalence studies of this kind [42,58]. Unlike the Pearson and Spearman correlation coefficients, the ICC_agreement_ is more appropriate for assessing agreement because it considers not only chance errors but also systematic errors [56,59]. ICC is expressed as a value between 0 and 1, with values >0.70 indicating adequate reliability [56,58]. The internal consistency between the paper and smartphone versions of each questionnaire was calculated using Cronbach’s alpha and McDonald’s omega. Furthermore, 95% confidence intervals (CIs) for these indices were calculated; values of Cronbach’s alpha and McDonald’s omega were denoted as 0–1. The alpha and omega values increase with the degree of correlation between the objects [60]. Good internal consistency is defined as Cronbach’s alpha and McDonald’s omega values of 0.7 or above [59,60]. In addition, linear mixed models (LMM) were used to confirm the carryover effect of each scale score [61]. In the LMM, the questionnaire administration format (paper or smartphone version), order of administration (paper or smartphone version first), and interaction between questionnaire administration format and order of administration are considered fixed-effect factors, whereas participants were considered random-effect factors. Statistical significance was set at *p* < 0.05 with a two-tailed test. All analyses were performed in R (version 4.0.2 for Windows; The R Project for Statistical Computing; Vienna, Austria).

## 3. Results

### 3.1. Characteristics of the Study Participants

Of the 100 participants who met eligibility, 100 completed the paper and smartphone versions of the questionnaire and provided complete data. In the paper-first group, 50 participants first completed a paper-version questionnaire. In the smartphone-first group, 50 participants first completed the smartphone version questionnaire. The mean age of the study participants was 19.86 years (SD = 1.08, 23% male), 9 (9%) had a drinking habit, 1 (1%) had a smoking habit, and 37 (37%) had an exercise habit (Table 1).

### 3.2. Mean and LMM Results

The mean values for each group and the LMM results are shown in Table 2. The interaction of questionnaire format and order of administration on the CES-D score was not significant (*p* = 0.96; 95% CI −1.71 to 1.79). The interaction of a questionnaire format and order of implementation on GAD-7 scores was not significant (*p* = 0.96; 95% CI −0.82 to 0.78). The interaction of a questionnaire format and order of implementation on the K6 score was not significant (*p* = 0.17; 95% CI −1.31 to 0.23). Based on these results, no carryover effects were observed.

### 3.3. Equivalence

The ICC_agreement_ values between the paper and smartphone versions of the CES-D, GAD-7, and K6 scores were 0.76 (95% CI 0.66–0.83), 0.68 (95% CI 0.59–0.77), and 0.83 (95% CI 0.75–0.88), respectively (Table 3).

### 3.4. Internal Consistency

Cronbach’s alpha values for the CES-D score were 0.82 (95% CI 0.77–0.87) and 0.81 (95% CI 0.75–0.86) for the smartphone and paper versions, respectively. Cronbach’s alpha values for the GAD-7 score were 0.80 (95% CI 0.75–0.86) and 0.80 (95% CI 0.75–0.86) for the smartphone and paper versions, respectively. Cronbach’s alpha values for the K6 score were 0.88 (95% CI 0.84–0.92) and 0.82 (95% CI 0.77–0.88) for the smartphone and paper versions, respectively (Table 4). McDonald’s omega values for the CES-D score were 0.83 (95% CI 0.75–0.87) and 0.81 (95% CI 0.74–0.86) for the smartphone and paper versions, respectively. McDonald’s omega values for the GAD-7 score were 0.83 (95% CI 0.76–0.87) and 0.84 (95% CI 0.72–0.91) for the smartphone and paper versions, respectively. McDonald’s omega values for the K6 score were 0.87 (95% CI 0.84–0.92) and 0.83 (95% CI 0.76–0.88) for the smartphone and paper versions, respectively (Table 4).

## 4. Discussion

This study evaluated the equivalence of the embodiments to the CES-D, GAD-7, and K6 evaluated in smartphone and paper versions. The results suggest that CES-D and K6 have good equivalence, with ICC_agreements_ of 0.76 and 0.83, respectively. Cronbach’s alpha values of the smartphone versions of CES-D and K6 were 0.82 (95% CI 0.77–0.87) and 0.88 (95% CI 0.84–0.92), respectively, indicating that they not only have good internal consistency but also comparable internal consistency to the paper versions of CES-D (0.81; 95% CI 0.75–0.86) and K6 (0.82; 95% CI 0.77–0.88). McDonald’s omega values for the smartphone versions of CES-D and K6 were 0.83 (95% CI 0.75–0.87) and 0.87 (95% CI 0.84–0.92), respectively, indicating that they not only have good internal consistency but also comparable internal consistency to the paper versions of CES-D (0.81; 95% CI 0.74–0.86) and K6 (0.83; 95% CI 0.76–0.88). The results suggest that the smartphone versions of the CES-D and K6 produce comparable self-assessments as the paper versions of the CES-D and K6. Previous studies have suggested that both ICC and Cronbach’s alpha should be at least 0.7 for group-level use and 0.85–0.95 for individual-level use [42]. Considering the ICC and Cronbach’s alpha criteria, the smartphone versions of CES-D and K6 are at least considered suitable for use at the group level. In other words, the smartphone versions of the CES-D and K6 may not be suitable for use on an individual level. However, it is crucial to remember that the ICC_agreement’s_ 95% CI for K6 was 0.75–0.88 and for CES-D was 0.66–0.83. This 95% CI indicates that, with a 95% probability, the true value of ICC_agreement_ for CES-D is 0.83 in the best case and 0.66 in the worst case [62]. Therefore, while the smartphone and paper versions of the CES-D reveal better agreement, they may also indicate lower agreement, below the threshold of 0.7, which is considered good. However, the ICC being below 0.7 may not necessarily be due to a low degree of agreement on the scale but also to issues of study design, such as low inter-subject variability sampled and sample size [63]. The low variability among sampled patients probably had an impact on the accuracy of the ICC_agreement_ estimations because our study had a large enough sample size to assess the ICC suggested by the Consensus-based Guidelines for the Selection of Health Measuring Instruments initiative [63]. We were restricted to a relatively young population (18–22 years old) in our sample. As a result, further investigation in a broader age population is required to provide more accurate estimates of ICC_agreement_ and its 95% CI.

The Cronbach’s alpha for the GAD-7 on smartphones was 0.80 (95% CI 0.75–0.86), indicating that it has the same internal consistency as the GAD-7 on paper (0.80; 95% CI 0.75–0.86). McDonald’s omega values for the GAD-7 on a smartphone were also 0.83 (95% CI 0.76–0.88), and they were 0.83 (95% CI 0.76–0.88) for the GAD-7 on paper, indicating strong internal consistency. However, the ICC_agreement_ for GAD-7 was 0.68 (95% CI 0.59–0.77), suggesting a low concordance between the smartphone and paper versions. This low ICC_agreement_ could be attributed to the changes following the transition from the paper version to the smartphone version. In this study, participants scrolled the screen to answer the items in each of the smartphone versions of the questionnaire. In addition, the questions and their response items were displayed in different positions in the paper and smartphone versions. These changes are defined as a moderate level of modification in the ISPOR guidelines, which is the level of modification that requires equivalence assessment [42]. In GAD-7, these changes from paper to smartphone versions may not have been suitable. Future studies should create a smartphone version of the GAD-7 with a display format more similar to the paper version to evaluate equivalence. It is also essential to note that the 95% CI for ICC_agreement_ in GAD-7, as in CES-D, was 0.59–0.77. This 95% CI means that the true value of ICC_agreement_ for GAD-7 is 0.77 in the best case and 0.59 in the worst case, with a 95% probability [62]. As a result, even while the GAD-7 on a smartphone or piece of paper would finally surpass the 0.7 criterion, they might still exhibit inferior agreement. However, even in the ICC_agreement_ for the GAD-7, the effect of the low sample variability in this study cannot be ignored [63]. Hence, similar to the CES-D, more research in a larger age range is required to more precisely estimate the ICC_agreement_ and its 95% CI.

As far as we could find, no studies have tested the equivalence of the electronic and paper versions of the K6 and GAD-7. However, previous studies have examined the equivalence of electronic and paper versions of the CES-D. A study of 2400 teachers in Taiwan, which tested the equivalence of the Internet-based CES-D and paper-based CES-D, found little difference in potential means and concluded that Internet-based CES-D is a promising alternative to paper-based CES-D [53]. In addition, the equivalence of the paper- and tablet-based methods was tested in 79 patients with low back pain, and the ICC was 0.75 (0.64–0.83), which is comparable to our results [52]. On the contrary, previous study have tested the equivalence of PC- and paper-based CES-Ds and suggested correlation coefficients ranging from 0.96 [64]. However, the Pearson and Spearman correlation coefficients are not extremely rigorous parameters for assessing equivalence because they do not account for systematic errors [42,59]. Considering the characteristics of the results of these previous studies and the potential advantages of smartphones (easy and ubiquitous accessibility), at least a smartphone version of CES-D may be a promising alternative strategy for PC- and tablet-based CES-D.

This study has several limitations. First, the study participants were a relatively young population, aged 18–22 years. Therefore, the results of this study may not apply to other age groups. Second, the influence of the carryover effects cannot be ignored. In a crossover design, a carryover effect may occur if the interval between the first and second evaluations is short. We tried to reduce the carryover effect as much as possible by keeping the interval between the first and second evaluations to 1 week. In fact, no statistically significant differences in the carryover effects were found in this study. However, given the lack of consensus on the ideal implementation interval when testing the equivalence of PROs [65], the influence of carryover effects must be carefully considered. Third, the smartphone and paper versions of the PROs were administered in the same room under the supervision of the researcher. If participants responded to the smartphone version of the PRO without meeting the researcher face to face, they may have been more anonymous than in our study and could have responded in a more natural setting. Therefore, the presence or absence of a supervisor and the effect of locations such as the clinic or home setting, should be fully considered. On the contrary, responding in the same room with the researcher made it possible to prevent omissions in the paper version and control for test conditions that would reduce the general likelihood of noise, distraction, mood fatigue, etc. [66]. Fourth, due to the difficulty of the participant burden in completing the questions, this study did not examine cognitive debriefing or usability testing, which are classified as minor alterations. Future studies should incorporate cognitive debriefing and usability testing of the smartphone versions of the CES-D, GAD-7, and K6, as these characteristics may considerably alter their usefulness in research and clinical contexts. Fifth, the smartphone versions of the CES-D, GAD-7, and K6 employed in this study could not be completed until all items were answered. The equivalency results reached in this study may have been impacted if participants were made to complete tasks they could have skipped in the paper version. Consequently, the effect of forced responses in the smartphone version of this study should be properly considered. Future studies should explore the equivalence of the paper and smartphone versions of the CES-D, GAD-7, and K6 by including a “choose not to answer” or “skip question” option. Sixth, participants replied to the CES-D, GAD-7, and K6 in that order on both the paper and smartphone versions of the survey. Thus, it was impossible to rule out the impact of ordering effects. As a result, the effects of order effects should be taken into consideration while interpreting the findings of this study.

## 5. Conclusions

This study demonstrates the equivalence of the paper and smartphone versions of the CES-D and K6. Accordingly, both the CES-D and K6 scales are appropriate for use in a smartphone version, which could be applied to clinical and research settings in which paper and smartphone versions could be selected as needed. However, the paper and smartphone versions of the GAD-7 should not be used interchangeably, as the paper and smartphone versions did not show equivalence because of low ICC_agreement_; thus, further research is needed.

## Figures and Tables

**Table 1:** Baseline characteristics of the two groups.

	Total (*n* = 100)	Paper First (*n* = 50)	Smartphone First (*n* = 50)
Characteristics			
Age (years)	19.86 (1.08)	19.88 (1.02)	19.84 (1.15)
Sex			
Male	23 (23%)	7 (14%)	16 (32%)
Female	77 (77%)	43 (86%)	34 (68%)
Drinker			
Yes	9 (9%)	5 (10%)	4 (8%)
No	91 (91%)	45 (90%)	46 (92%)
Smoker			
Yes	1 (1%)	0 (0%)	1 (2%)
No	99 (99%)	50 (100%)	49 (98%)
Exercise habits			
Presence	37 (37%)	15 (30%)	22 (44%)
Absence	63 (63%)	35 (70%)	28 (56%)

Data are means (standard deviation) or numbers (%).

**Table 2:** Means (SD) and LMMs results.

	Total (*n* = 100)	Paper First (*n* = 50)	Smartphone First (*n* = 50)	LMM			
Outcomes	Mean (SD)	Mean (SD)	Mean (SD)	Effect	Estimate	*p*	95% CI
CES-D							
Paper	11.07 (6.34)	10.92 (5.42)	11.22 (7.20)	Format	−0.44	0.49	−1.68 to 0.80
Smartphone	11.05 (6.50)	10.48 (6.18)	11.62 (6.82)	Order	0.7	0.59	−1.82 to 3.22
				Interaction	0.04	0.96	−1.71 to 1.79
GAD-7							
Paper	2.19 (2.66)	1.88 (2.18)	2.50 (3.06)	Format	0.36	0.22	−0.21 to 0.93
Smartphone	2.20 (2.49)	2.24 (2.70)	2.16 (2.35)	Order	0.28	0.59	−0.73 to 1.29
				Interaction	−0.02	0.96	−0.82 to 0.78
K6							
Paper	2.40 (3.09)	2.38 (3.10)	2.42 (3.12)	Format	0.36	0.279	−0.19 to 0.91
Smartphone	2.67 (3.55)	2.74 (3.65)	2.60 (3.49)	Order	0.22	0.74	−1.09 to 1.53
				Interaction	−0.54	0.17	−1.31 to 0.23

CES-D: Center for Epidemiologic Studies Depression Scale; GAD-7: Generalized Anxiety Disorder-7; K6: Kessler Screening Scale for Psychological Distress; SD: standard deviation; LMM: linear mixed models.

**Table 3:** Intragroup ICC (95% CI) for the CES-D, GAD-7, and K6.

Outcomes	ICC_agreement_	95% CI
CES-D	0.76	0.66–0.83
GAD-7	0.68	0.59–0.77
K6	0.83	0.75–0.88

CES-D: Center for Epidemiologic Studies Depression Scale; GAD-7: Generalized Anxiety Disorder-7; K6: Kessler Screening Scale for Psychological Distress; ICC: intraclass correlation coefficient; CI: confidence interval.

**Table 4:** Internal consistency for CES-D, GAD-7, and K6.

Outcomes	Cronbach’s Alpha	95% CI	McDonald’s Omega	95% CI
CES-D				
Paper	0.81	0.75–0.86	0.81	0.74–0.86
Smartphone	0.82	0.77–0.87	0.83	0.75–0.87
GAD-7				
Paper	0.80	0.75–0.86	0.84	0.72–0.91
Smartphone	0.80	0.75–0.86	0.83	0.76–0.87
K6				
Paper	0.82	0.77–0.88	0.83	0.76–0.88
Smartphone	0.88	0.84–0.92	0.87	0.84–0.92

CES-D: Center for Epidemiologic Studies Depression Scale; GAD-7: Generalized Anxiety Disorder-7; K6: Kessler Screening Scale for Psychological Distress.

## Data Availability

The datasets used and/or analysed during the current study available from the corresponding author on reasonable request.
